# Subcutaneous Immunoglobulin Therapy in the Chronic Management of Myasthenia Gravis: A Retrospective Cohort Study

**DOI:** 10.1371/journal.pone.0159993

**Published:** 2016-08-04

**Authors:** P. R. Bourque, C. E. Pringle, W. Cameron, J. Cowan, J. Warman Chardon

**Affiliations:** 1 The Ottawa Hospital, Department of Medicine, Division of Neurology, Ottawa, Canada; 2 University of Ottawa, Faculty of Medicine, Ottawa, Canada; 3 The Ottawa Hospital, Department of Medicine, Division of Infectious Diseases, Ottawa, Canada; 4 The Ottawa Hospital Research Institute, Ottawa, Canada; 5 Children’s Hospital of Eastern Ontario, Division of Genetics, Ottawa, Canada; Istanbul University, TURKEY

## Abstract

**Background:**

Immunoglobulin therapy has become a major treatment option in several autoimmune neuromuscular disorders. For patients with Myasthenia Gravis (MG), intravenous immunoglobulin (IVIg) has been used for both crisis and chronic management. Subcutaneous Immunoglobulins (SCIg), which offer the advantage of home administration, may be a practical and effective option in chronic management of MG. We analyzed clinical outcomes and patient satisfaction in nine cases of chronic disabling MG who were either transitioned to, or started de novo on SCIg.

**Methods and Findings:**

This was a retrospective cohort study for the period of 2015–2016, with a mean follow-up period of 6.8 months after initiation of SCIg. All patients with MG treated with SCIg at the Ottawa Hospital, a large Canadian tertiary hospital with subspecialty expertise in neuromuscular disorders were included, regardless of MG severity, clinical subtype and antibody status. The primary outcome was MG disease activity after SCIg initiation. This outcome was measured by 1) the Myasthenia Gravis Foundation of America (MGFA) clinical classification, and 2) subjective scales of disease activity including the Myasthenia Gravis activities of daily living profile (MG-ADL), Myasthenia Gravis Quality-of-life (MG-QOL 15), Visual Analog (VA) satisfaction scale. We also assessed any requirement for emergency department visits or hospitalizations. Safety outcomes included any SCIg related complication. All patients were stable or improved for MGFA class after SCIg initiation. Statistically significant improvements were documented in the MG-ADL, MG-QOL and VAS scales. There were no exacerbations after switching therapy and no severe SCIg related complications.

**Conclusions:**

SCIg may be a beneficial therapy in the chronic management of MG, with favorable clinical outcome and patient satisfaction results.

## Introduction

Myasthenia gravis (MG) is the most common disorder of the neuromuscular junction, with a prevalence of 20/100 000 in various populations [1]. Its pathogenesis involves complement fixing antibodies directed against acetylcholine receptors, muscle-specific tyrosine kinase or low-density lipoprotein receptor–related protein 4. Although conventional oral immunosuppressive medications have remained the mainstay of therapy, intravenous immunoglobulin therapy (IVIg) has been used increasingly, both acutely for the management of exacerbations and chronically for refractory MG [2]. There has been considerable interest in recent years in the chronic subcutaneous route of administration of immunoglobulins (SCIg), first in immunodeficiency syndromes [3], and subsequently in several types of inflammatory neuromuscular disorder [4]. We report the experience our center, The Ottawa Hospital has had using SCIg for nine patients with chronic MG, refractory to conventional oral immunotherapy. This cohort covers a wide spectrum of clinical subtype, severity and antibody status. Our objective was to compare the clinical response before and after initiation of SCIg, using standardized assessment scales. For patients previously on IVIg, we also compared immunoglobulin dosage.

## Methods

This retrospective study was approved by the Ottawa Hospital Research Ethics board. All patient records and information were anonymized and de-identified prior to analysis. Cases were identified from The Ottawa Hospital (TOH) Neuromuscular Disease Database. All patients with MG transitioned to SCIg between January 2015 and December 2015 were recruited and agreed to participate. The diagnosis of MG was supported by established criteria, including a characteristic clinical course, electrophysiological abnormalities (repetitive stimulation, single fiber electromyography) or the presence of myasthenia-associated auto-antibodies. Nine consecutive patients with MG were included for this retrospective case series study. Human Immune Globulin subcutaneous (Hizentra, 20g/100 ml, CSL Behring) was administered in the anterior abdomen, with an initial total infusion rate was no more than 20–30 ml /hour, though this was gradually increased up to 50 ml/hour. For the six patients already on IVIg the initial target weekly amount was calculated at 120% of the equivalent IVIg weekly dose. For the remaining three patients, SCIg was empirically started at 20g per week. Subsequent dose adjustments were prescribed according to clinical response. All participants had been screened for commonly accepted exclusion criteria for the use of immunoglobulins, including: renal insufficiency, abnormal liver function (transaminases elevation greater than 2.5 the upper limit of normal), history of thrombotic event in the past year or established high risk of thrombosis. There were no exclusions from the sample of nine, as a result of this screening.

The primary outcome was MG disease activity after SCIg initiation, measured by 1) the Myasthenia Gravis Foundation of America (MGFA) clinical classification, 2) subjective scales of disease severity including the Myasthenia Gravis activities of daily living profile (MG-ADL) [5] and the Myasthenia Gravis Quality-of-life (MG-QOL 15) questionnaire [6]. As MG is an inherently variable disease, patients were asked to subjectively average their symptoms for a period of one month at two time points: before institution of SCIg, and the month after a stable dosage was achieved. Questionnaires were administered in sequence after instruction for a before- and after-SCIg comparison, at a single point after establishment of home SCIg self-treatment. The MG-ADL is a validated scale scored 0–3 for 8 specific symptoms characteristic of myasthenic weakness, namely: talking, chewing, swallowing, breathing, ability to brush teeth or comb hair, ability to arise from a chair, diplopia and ptosis. The MG-QOL 15 has been designed to capture the “role-physical” and “social functioning” subscale of the Short Form (36) Health Survey. Additionally, a Visual Analog Scale (VAS) was used to capture overall subjective response to therapy for the same time points. The scale ranged from zero (worst possible control of MG) to ten (best possible control of MG). Safety outcomes included the occurrence of any emergency department visit or hospitalization for MG exacerbation after initiation of SCIg, as well as significant infusion-related complications, based on the review of hospital files and direct structured interview of all patients by one neuromuscular expert (PB).

## Results

During the study period, four patients declined switching from IVIg to SCIg, two citing their phobia of self-injection, two expressing their satisfaction with their IVIg program. All nine MG patients started on SCIg between January 2015 and December 2015 were included in this survey and analysis. Six patients were noting benefit from their IVIg regimen and were switched to SCIg for the convenience of home self-administration. Two patients had previously discontinued IVIg because of allergic side-effects to IVIg. One patient had transiently received IVIg 10 years previously and had been lost to neurological follow-up. She was experiencing increasing disability from MG, but was no longer able to attend hospital appointments. Thus three patients newly started SCIg treatment without a prior period of IVIg treatment for comparison. All nine study patients had been treated with prednisone and pyridostigmine earlier in the course of MG. All had been tried on at least one additional immunosuppressive medication (azathioprine in 8 patients, mycophenolate mofetil in 2 patients). Three patients had demonstrated intolerance to azathioprine. Six patients had eventually stopped prednisone altogether. Four of these had achieved satisfactory control with the combination of azathioprine and immunoglobulin therapy. The other two did not want to take oral immunosuppressive treatment, for reasons of intolerance or fear of side-effects. The mean age was 50.6 years (range 21–84). Five patients were AChR Antibody positive. One patient was AChR and MUSK antibody negative. Two other AChR antibody negative patients had never been tested for MUSK status, because they were diagnosed 16 and 17 years ago. Three patients had been found to have thymoma and four patients had a thymectomy. Five other patients had elected not to undergo thymectomy after a discussion with their treating neurologist and the thoracic surgery team. Disease duration ranged from 1–31 years (mean 11.8 years). Seven patients remained on additional symptomatic or immunomodulating therapy during SCIg treatment. MG severity was graded as mild to moderate (grades II-III) by MGFA clinical classification (IVIg) (Table 1).

**Table 1:** Patient Characteristics.

Patient	Age, sex	Antibody Status A = AChRA, M = MUSK	Thymoma (Yes, No)	Thymectomyes, (Y (Yes, no)	Concurrent MG therapy	Interval: clinical MG onset to SCIg (yrs)
**1**	48 F	A-, M-	N	N	AZT 200 mg/d, Pred 20 mg/d, Pyrido 240 mg/d	1.7
**2**	49 F	A+	N	N	Nil*	4
**3**	49 F	A-	N	Y	AZT 125mg/d, Pyrido 240mg/d	17
**4**	33 F	A+	Y	Y	AZT 200 mg/d Pyrido 360mg/d	2.8
**5**	21 F	A+	N	N	AZT 200mg/d	5.5
**6**	49M	A+	Y	Y	AZT 150 mg/d, Pred 17.5mg/d, Pyrido 420mg/d	26
**7**	63 M	A+	N	N	Pred 5mg/d	2
**8**	60 F	A+	Y	Y	AZT 200mg/d	31
**9**	83 F	A-	N	N	Nil*	16

MGFA: Myasthenia Gravis Foundation of America, AChRA: Acetyl choline receptor antibody, MUSK: Muscle specific kinase antibody, Pred: Prednisone, Pyrido: pyridostigmine, AZT: azathioprine.

* Immunosuppressive medications discontinued prior to SCIg because of intolerance, lack of effect or patient choice.

### MG Activity following SCIG initiation

At a mean time point of 6.8 months (range 2–11) after initiation of SCIg, all patients had a MGFA clinical classification that was stable or improved (Table 2). Using a paired two-tailed T test, there was a statistically significant trend favoring SCIg therapy for all three scale measurements: MG-ADL (p = 0.005), MG-QOL (p = 0.003) and VAS (p = 0.005). For the subgroup of 6 patients previously on IVIg, a statistically significant benefit was demonstrated for MG-ADL (p = 0.049) and MG-QOL (p = 0.008) and for VAS (p = 0.013). For the MG-ADL, only 4 of 9 patients had an improvement of greater than 3 points, which is the limit for clinical significance. Four patients specifically reported achieving a more steady control of myasthenic symptoms compared to IVIg treatment related fluctuations, typically those “breakthrough” symptoms in the week prior to scheduled monthly IVIg.

**Table 2:** Treatment and assessment data.

Patient	SCIg weekly dosage ^1^	IVIg weekly dosage comparison ^2^	Average time (per infusion, hours)	MGFA Class		MG QOL 15 ^3^			MG-ADL ^4^		VAS ^5^	
				Pre SCIg	On SCIg	Pre SCIg	On SCIg		Pre SCIg	On SCIg	Pre SCIg	On SCIg
**1**	20 g/2	17.5	1	III	I	25	14		8	3	6	8
**2**	30 g/2	18.7	1.5	II	II	15	10		12	9	6.5	8
**3**	30 g/2	18.7	1.5	III	II	6	0		1.5	1.5	7	10
**4**	16 g/1	16,3	1.25	II	II	9	8		2	2	9	9
**5**	20 g/2	13.8	1.5	II	I	11	5		2	0	7	8
**6**	30 g/2.5	25	1.25	III	III	17	12		10	8	7	9
**P valuePt 1–6**						0.008			0.049		0.013	
**7**	40 g/3	N/A	2	III	III	36		28	10.5	8	2.1	8.1
**8**	20 g/2	N/A	2	III	IIb	30		13	8	4	3.5	9.5
**9**	20 g/2	N/A	1.5	III	III	35		33	15	14	3	5.5
**Mean**	25.1	18.3	1.5			20.4		13.7	7.7	5.6	5.8	8.2
**P valuePt 1–9**						0.003			0.005		0.005	

^1^: Total weekly immunoglobulin (g) / number of infusion days

^2^: IVIg dosage, expressed as weekly amount

^3^: Myasthenia Gravis Quality of Life questionnaire

^4^: Myasthenia Gravis Activity of daily living profile

^5^: Visual analogue scale (0 = worst possible control of MG, 10 = best possible control of MG). P value calculated using a two-tailed paired T test. A statistically significant benefit was obtained on all three scales for the entire group (1–9) and for the subgroup (1–6) initially treated with IVIg.

### Dosage and Adverse events with SCIG

The SCIg dosage was empirically titrated to optimal clinical response, starting with a 120% conversion from IVIg when applicable (6 patients). The SCIg weekly immunoglobulin doses ranged from 16 to 40 g (mean 25.1g, SD 7.8). A direct dosage comparison could be made for the 6 patients who were switched from IVIg to SCIg. This cohort reached weekly optimal SCIg dose of 24.3 g (or 0.39 g/kg) representing 133% of the average weekly amount of 18.3 g (or 0.29 g/kg) during prior IVIg treatment. No patient complained of systemic symptoms attributable to SCIg. Most patients did mention mild subcutaneous tenderness or pruritus on the day of infusion, with frequent circumscribed bruising, which were not of concern. Patient #3 reported more prominent ecchymoses; she was one of two patients concurrently receiving chronic oral steroid treatment. There were no emergency room visits or ICU admissions after commencement of SCIg.

## Discussion

We describe a successful transition to SCIg, validated by MGFA classification and by functional scales (MG-ADL, MG-QOL) in 9 patients with refractory chronic MG.

The management of acquired autoimmune myasthenia gravis remains challenging, as a majority of patients require long-term therapy. In one large Italian series [7], complete stable remission was observed in only 3.6% of MUSK-positive, 22.2% of AChR positive and 21.9% of double-negative patients. In a series from Duke University, remission with cessation of medications was achieved in only 20% of all AChR positive and 7% of thymoma patients [8]. Immunoglobulin therapy is expected to play an increasing role, particularly for patients showing a partial response to standard long-term immunosuppressive medications or who cannot tolerate associated side-effects [9].

IVIg is increasingly used in the management of acute MG exacerbations, comparable in efficacy and tolerability to plasma exchange (Class II evidence) [10,11]. However, the benefit of IVIg in moderately severe but stable MG has not yet been documented in large randomized controlled trials [12,13] except for a favorable comparison to plasmapheresis in the setting of juvenile myasthenia gravis [14]. In an open-label study of 10 patients with severe generalized myasthenia gravis with acute deterioration unresponsive to conventional therapies, Achiron [15] reported that IVIg 400mg/kg for 5 consecutive days was effective in inducing rapid improvement. Furthermore, the same patients maintained satisfactory remission with further infusions of 400mg/kg every 6 weeks over the following year.

Self–administration of SCIg at home was first introduced for patients with primary immunodeficiency, and offers an effective, practical and well tolerated alternative to IVIg [16,17]. The main reported advantages of SCIg are increased patient autonomy, lower rate of treatment-related allergic or toxic reactions, and decreased costs related to hospital resources [17,18]. IVIg and SCIg have been found to be equally effective for patients with primary immune deficiencies [19]. Studies of health-related quality of life and treatment satisfaction show a significant patient preference both for the subcutaneous route over IV administration (81%) and the option of home therapy (90%) [20].

Many studies have suggested that SCIg may be efficacious and well tolerated in several other chronic immune neuromuscular disorders such as CIDP [21,22,23], multifocal motor neuropathy [23,24,25,26] and inflammatory myopathy [27,28,29,30]. The literature so far has however consisted of open-label, uncontrolled or retrospective studies. This present case series documents successful transition to SCIg in 9 patients with a wide spectrum of chronic MG, including antibody positive (6/9) and negative (3/9), thymomatous (3/9), as well as a wide range of disease duration (from 1.7 to 31 years) and clinical subtypes (MGFA II, IIb, and III). All patients achieved a stable or improved MGFA classification and there were statistically significant trends favoring SCIg in the MG-ADL, MG-QOL 15 and VAS overall satisfaction scales. To our knowledge, there has been only one previous report of a patient with chronic MG treated with of SCIg with stable symptoms for 8 years, and a 20% dose reduction on SCIg compared to IVIg [4].

In this current cohort of patients with chronic MG, of the six patients who were transitioned from IVIg, four reported that SCIg helped them achieve a more constant control of myasthenic weakness. This benefit may be attributable to achieving more steady serum immunoglobulin levels with SCIg, avoiding the trough expected at the end of the inter-infusion interval with monthly IVIg [31] and the sometimes familiar breakthrough of MG symptoms in the week or days before periodic IVIg dosing. The administration of SCIg requires fractionating the monthly dose of Ig into smaller portions of weekly or twice weekly doses, which decreases the variability in serum IgG levels and maintains more consistent serum IgG levels [32]. However, in acute myasthenic exacerbations, rapidly achieved peak serum Ig concentrations may be desired, and the lower, more frequent dosing of SCIg may not be beneficial. There is evidence to suggest that IVIg may offer a benefit comparable to plasmapheresis in this particular setting [10]. It is of note that the introduction of SCIg did not lead to a reduction in concurrent immunotherapy.

None of our patients experienced systemic effects commonly attributed to high peak levels of immunoglobulins with IVIg, such as fever, headache, hypertension or thromboembolic events [31]. In the subset of 6 patients where a dosage comparison was applicable, we found that the SCIg dosage was significantly higher than previous IVIg use, by a factor of 132.3%. Subcutaneous absorption is not expected to achieve the same systemic distribution as intravenous infusion. A pharmacokinetic study of SCIg 20% [33] found that the dose-adjustment coefficient, compared to IVIG, ranged from 1.26 to 1.87, with a mean of 1.53. In a US cohort of patients with primary immunodeficiency [34], a conversion dose factor of 1:1.5 was recommended, estimating that higher doses achieve better biologic effect and thus reduce utilization of resources. The titration to optimal clinical response required an average dose increase of 120% to 132.3%, from the initial empirical dose, usually within the first month. Compared to hospital based prescribed monthly IVIg, SCIg self-administration may also encourage patients to act as more autonomous stakeholders, who more readily advocate for dosage increases. Given the high cost of Ig therapy, if IVIg or SCIg are to be used more widely in chronic MG, consideration will need to be given to cost analysis. Immunoglobulin therapy is undoubtedly more expensive than all other conventional oral immunosuppressive medications such as prednisone, azathioprine, mycophenolate or cyclosporine. However, the indirect costs and risks associated with significant long-term complications of such medications must however also be taken into consideration.

There are limitations to this retrospective self-control case-series analysis, as in other reports of open design [35]. This case series is small, and reflects the experience of a few neurology practices in a single centre. Four of thirteen candidates approached during had declined to try SCIg. However, there was no selection bias among SCIg-treated patients evaluated, as all nine were included in evaluation. A potential limitation of this study is recall bias for subjective scales of well-being and function comparing MG-related symptoms before and after institution of SCIg. The study design however allowed patients to make a more direct, one-time subjective before-and-after comparison over the transition in their treatment plan.

Patients were mostly keen to improve myasthenic symptoms and to be more autonomous from regular hospital visits. The probability of a true benefit is strengthened by the fact that all three scales (MG-ADL, MG-QOL15, VAS) showed a consistent direction of effect. As well, no patient requested to return to IVIg treatment after switching to SCIg.

## Conclusions

The efficacy of SCIg has been demonstrated in other treatment-refractory autoimmune neuromuscular disorders. We report on the benefit, safety and tolerability of SCIg for chronic MG control for the largest reported cohort for patients with moderate disease and wide spectrum of MG severity, clinical subtype and antibody status. The role of SCIg in chronic management for MG warrants further exploration with larger prospective studies, which would include additional objective and reproducible measures of disease activity such as the Quantitative Myasthenia Gravis score [36]. Consideration will also have to be given to an economic analysis of SCIg in relation to conventional oral immunotherapy and IVIg.
